# Supplementary material for: Brazilian Validation of the European Organisation for Research and Treatment of Cancer (EORTC) Quality of Life Group (QLG) Computerised Adaptive Tests (CAT) Core
Source: Curr Oncol. 2021 Aug 31;28(5):3373–83. doi: 10.3390/curroncol28050291 (PMC8482129; doi:10.3390/curroncol28050291)
Supplement: Supplementary file 1 [file curroncol-28-00291-s001.zip › curroncol-1227041-supplementary.pdf]

Current Oncology

Supplementary Materials: Brazilian Validation of the European Organisation for Research and Treatment of Cancer (EORTC) Quality of Life Group (QLG) Computerised Adaptive Tests (CAT) Core

Gustavo Nader Marta, Tomás Y. T. de Souza, Alice R. N. de Souza e Silva, Ana Paula A. Pereira, David R. Ferreira Neto, Rie N. Asso, Fabiana A. M. Degrande, Guilherme Nader-Marta, Maurício F. da Silva, Rafael Gadia, Samir A. Hanna, Bernhard Holzner, Everardo D. Saad and Morten Aagaard Petersen

Table S1. – QLQ-C30 mean scores at T0 in Brazil and the difference between Brazilian and European mean scores.

Domain	Brazil	Brazil - Europe
**AP**	11.5	-12.0
**CF**	85.3	2.8
**CO**	14.3	-1.2
**DI**	6.0	9.0
**DY**	6.0	20.8
**EF**	74.7	69.5
**FA**	18.7	37.0
**FI**	17.9	12.1
**NV**	5.0	7.8
**PF**	82.4	78.1
**PA**	15.1	23.6
**RF**	85.2	70.0
**SF**	81.2	67.0
**SL**	27.2	28.3
**QL**	74.4	61.2

Note: AP lack of appetite, CO constipation, DI diarrhea, DY dyspnea, FI financial difficulties, SL insomnia, NV nausea/vomiting, PA pain, FA fatigue, CF cognitive functioning, RF role functioning, SF social functioning, EF emotional functioning, PF physical functioning and QL overall quality of life.

Table S2. – Mean relative validity/sensitivity (ability to detect cross-sectional differences) of the EORTC CAT Core compared to the QLQ-C30.

Domain	Brazil	Brazil-Europe	Minimum	Maximum
**AP**	1.18	0.09	0.56	1.72
**CF**	1.07	0.00	0.63	1.71
**CO**	1.14	-0.04	0.77	1.60
**DI**	1.27	-0.14	0.75	1.62
**DY**	4.58	3.32	0.84	36.24
**EF**	1.04	-0.07	0.60	1.60
**FA**	1.04	-0.01	0.72	1.30
**FI**	1.38	-0.16	0.64	4.33
**NV**	1.02	-0.11	0.38	1.48
**PA**	1.01	-0.12	0.75	1.30
**PF**	0.95	-0.04	0.60	1.38
**RF**	1.11	-0.26	0.74	1.75
**SF**	1.09	-0.06	0.79	1.53
**SL**	1.17	0.01	0.86	1.46
**Median**	1.10	-0.05	0.95	4.58

Note: AP lack of appetite, CO constipation, DI diarrhea, DY dyspnea, FI financial difficulties, SL insomnia, NV nausea/vomiting, PA pain, FA fatigue, CF cognitive functioning, RF role functioning, SF social functioning, EF emotional functioning and PF physical functioning.
